# A Case Report of Non-intentional Foreign Body Ingestion in an Elderly Patient

**DOI:** 10.7759/cureus.37684

**Published:** 2023-04-17

**Authors:** Omar G Alturkmani, Maysa M Al-Badawi, Shafeq G Alturkmani, Muhammad H Al-Midani, Shahd A Attar

**Affiliations:** 1 Gastroenterology, Digestive Disease and Nutrition Center, Burton, USA; 2 Gastroenterology, Digestive Disease and `Nutrition Center, Burton, USA; 3 Medicine, Alfaisal University College of Medicine, Riyadh, SAU; 4 Medicine, University of Hama, Hama, SYR

**Keywords:** foreign body ingestion and dementia, non-intentional foreign body ingestion, egd indication in foreign body ingestion, foreign body ingestion risk factors, foreign body ingestion in adults, foreign body ingestion treatment, foreign body ingestion complications, dysphagia in elderly

## Abstract

*Foreign body ingestion* is a common problem that can result in severe consequences. It occurs commonly in children and rarely in adults. High-risk adults include illicit drug users, prisoners, edentulous adults, alcoholics, psychiatric patients, adults with mental retardation, or those with decreased oral tactile sensation. In adults, most foreign body impactions are seen in patients with pre-existing pathologies, such as malignancy, achalasia, strictures, and esophageal rings. Complications that foreign bodies may cause in some cases are tracheoesophageal fistula, aorto-esophageal fistula, and intramural perforation. This case illustrates the importance of including foreign body ingestion in the differential diagnosis of dysphagia in high-risk groups, even when no clear history suggests this as a cause, which may decrease the complications.

## Introduction

Foreign body ingestion is a common problem that can result in severe consequences. It accounts for 1500 deaths in the United States annually [1]. It is defined as any object ingested intentionally or accidentally or materials ingested naturally when eating or taking medication, which can lead to complications such as impaction in special circumstances. Foreign bodies in the gastrointestinal tract are commonly seen in the emergency department, mostly among pediatrics [1]. Most foreign body ingestion in adults occurs accidentally but may result from contributory factors, such as alcohol consumption, edentulous state, mental retardation, and psychiatric disorders [1]. It is mainly seen in adults with pre-existing pathologies, such as strictures (about 37%), malignancy (about 10%), esophageal rings (about 6%), and achalasia (about 2% of cases) [2]. However, healthy adults with no risk factors are practically encountered unintentional foreign body ingestion [1].

According to a study by Adhikari et al. (2007), the most frequently ingested foreign bodies in adults and the elderly were meat bone at 76.1%, followed by a coin at 3.6% and dentures at 2.4%. Foreign bodies usually occur in the esophageal and cricopharyngeal regions, and most of these that pass beyond the esophagus will eventually pass through the intestinal tract. It is crucial to determine the degree of urgency and the best intervention modality once foreign body ingestion is diagnosed to reduce the risk of complications, such as intramural perforation, aortoesophageal fistula, subacute mediastinitis, tracheoesophageal fistula, and long term injury to the esophagus. The most common site foreign bodies were found in the esophagus at 62%, followed by the cricopharyngeal junction at 25% of cases and the pyriform sinus at 4.7% [3].

Management options include conservative treatment in 80% of cases, endoscopic intervention in 20%, and less than 1% will require surgery. Close observation is the treatment of choice for narrow (<2.5cm diameter), blunt, and short (<6cm) foreign bodies. Emergency esophagogastroduodenoscopy (EGD) is mandatory in complete occlusion of the esophagus with bolus impaction, sharp foreign bodies, and batteries. EGD within 12-24 hours is indicated in cases of magnets ingestion, foreign body >6cm in length, and other foreign bodies in the esophagus. However, elective EGD is required in foreign bodies >2.5cm in diameter and pre-pyloric foreign bodies. Surgical intervention is mandatory in cases of perforation and required in cases of failed endoscopy attempts and in the presence of complications that cannot be managed endoscopically [2].

## Case presentation

A 67-year-old male with dysphagia was referred to the gastroenterology clinic for removal of an unrecognized ingested foreign body seen in the stomach on an abdominal X-ray conducted on 1/12/2022. The patient was not aware of its ingestion. The patient has had progressive dysphagia to solids since 2019. He had a history of weight loss of 20 pounds over one year. An abdominal X-ray was done to rule out a foreign body. It was reported compared to a previous lumbar X-ray conducted on May 2020 for low back and hip pain. It showed a metallic object projecting over the abdomen of uncertain etiology, which could be related to an ingested foreign body. This patient has a medical history consistent with hypertension, subdural hematoma in 1986, hepatitis C treated eight years prior, dementia, peripheral neuropathy, bilateral hip arthritis, aortic rupture 30 years back, and dependent on opioids for 30 years. The patient smokes 5-6 cigarettes/day and has been smoking for approximately 40 years. The patient quit drinking alcohol 30 years back. He had a history of marijuana use a couple of times per week for the last fifty years.

An examination showed an alert and cachectic man with a body mass index of 18.3, and he was in no acute distress. An extremities examination showed muscle atrophy. A chest and abdominal examination were normal besides a right-sided graft (cadaver vascular graft passing underneath the skin).

**Figure 1:**
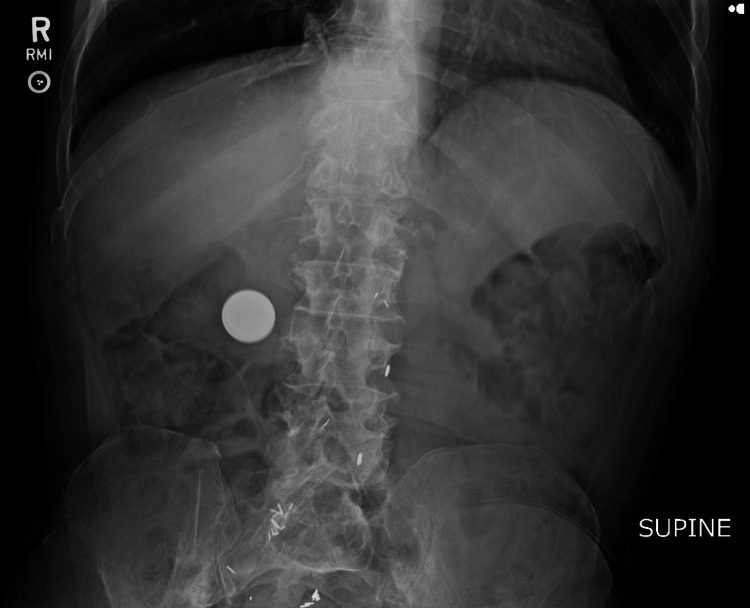
Abdominal X-ray Foreign body in the distribution of the distal stomach.

An abdominal X-ray showed a button battery foreign body in the distribution of the distal stomach (Figure 1). It appeared too large to pass out of the stomach as its position had not significantly changed compared with a lumbar X-ray conducted two years ago.

**Figure 2:**
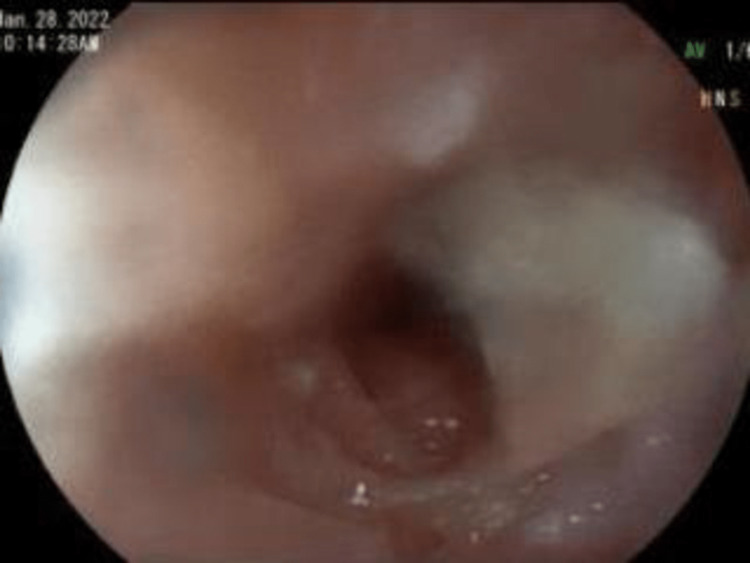
Esophagus Very tight distal esophageal stricture.

**Figure 3:**
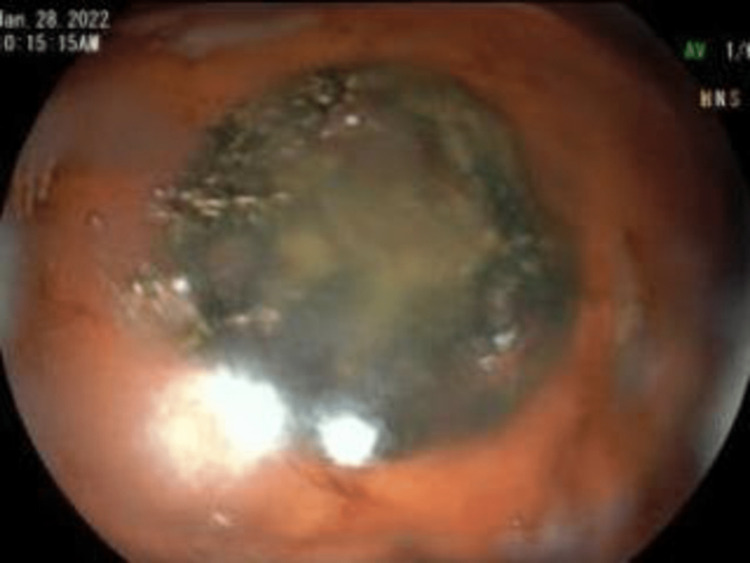
Foreign bodies in the stomach.

**Figure 4:**
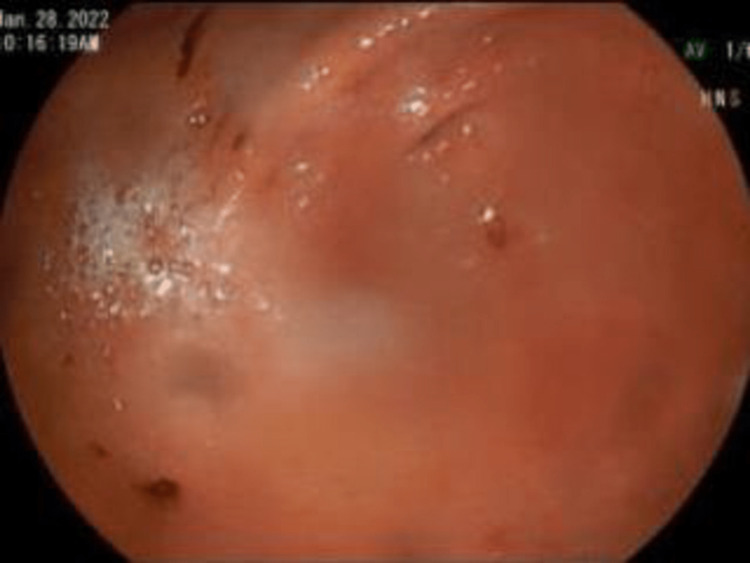
Antrum Erosive gastritis.

**Figure 5:**
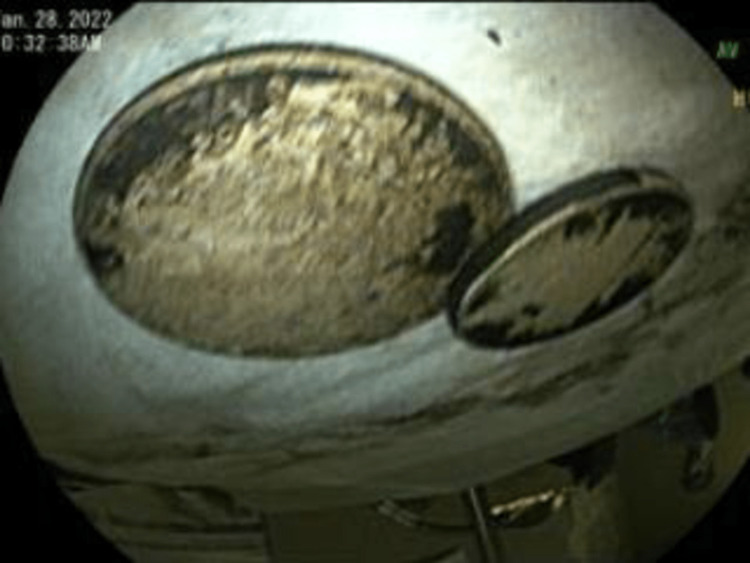
Foreign bodies (Removed)

Upper endoscopy with esophageal dilation showed very tight distal esophageal stricture (Figure 2), foreign bodies in the stomach (Figure 3), and erosive gastritis (Figure 4). Roth Net removed foreign bodies: one nickel coin and one quarter (Figure 5).

Two months after foreign body removal, the patient was contacted by telehealth, wherein he informed that his dysphagia had resolved after the procedure, and he had no abdominal pain.

## Discussion

Foreign body ingestion is a common clinical problem with potentially life-threatening complications. The estimated annual incidence in the United States alone is 120000 cases. Most of these cases occur in children, especially between 6 months and 3 years of age [4].

In adults, high-risk groups include those with acute intoxication, severe psychiatric disorders, mental retardation, or seeking a secondary gain (e.g., prisoners seeking referral to a medical facility) [4]. In addition, bolus ingestion in the elderly is common, especially in those who are edentulous and who cannot chew food properly, particularly meat. Moreover, most elderly patients have underlying pathologies causing feeding problems that need to be screened [3]. These pathologies include strictures, malignancies, achalasia, and esophageal rings [2].

Some foreign bodies which are sharp, large, and of specific types (e.g., batteries) may cause serious complications. These complications include impaction, erosion, mediastinitis, perforation, abscess, ulceration, empyema thoracis, oesophagoaortic fistula, and risk of death [3-4]. These complications mainly occur at sites of pathologic or physiologic narrowing or sharp angulation [4].

Management

In 80% of cases, foreign body ingestion is asymptomatic, and the foreign body passes without complications. However, endoscopic intervention is required in around 20% of cases, and surgical intervention is indicated in less than 1% of cases [2].

Conservative treatment

Since most swallowed foreign bodies pass the gastrointestinal tract without any problem, conservative treatment, including close observation, is indicated in most cases. This is the treatment of choice for narrow (<2.5cm diameter), blunt, and short (<6 cm) foreign bodies, especially once the foreign bodies have passed beyond the pylorus. The spontaneous passage is mostly expected within 4-6 days; however, it might take up to 4 weeks in rare cases [2].

Endoscopic intervention

Esophagogastroduodenoscopy (EGD) is indicated in removing upper gastrointestinal foreign bodies [2]. Indications for EGD are listed in Table 1.

**Table 1:** Indication for esophagogastroduodenoscopy and recommendations for immediate additional treatment [2] FB: Foreign bodies; EGD: esophagogastroduodenoscopy

Urgent need for endoscopy	Type of FB	Recommended treatment
Emergency EGD	Bolus impaction with complete occlusion of the esophagus	Inpatient/outpatient
	Sharp/pointed FB	Inpatient
	Batteries	Inpatient
EGD within 12–24 hours	Magnets	Inpatient
	FB >6 cm in length	Inpatient/outpatient
	Other FB in the esophagus	Inpatient/outpatient
Elective EGD	FB >2.5 cm diameter	Outpatient
	Prepyloric FB	Outpatient

Surgical treatment

In less than 1% of foreign body ingestion cases, surgical management is indicated. Surgical intervention's absolute indication would be in case perforation happened only. Relative indications include multiple failed endoscopic attempts and, in cases with complications that cannot be treated endoscopically [2].

## Conclusions

Foreign body ingestion, although rare in adults, should not be missed in high-risk individuals for foreign body ingestion presenting with dysphagia. Such patients include psychiatric patients, those with decreased oral tactile sensation, prisoners, illicit drug users, edentulous, intoxicated, alcoholics, and mentally disabled adults, or in those with pre-existing pathology for foreign body impaction. These pathologies include malignancy, achalasia, strictures, and esophageal rings. Including foreign body ingestion in these groups' differential diagnosis list of dysphagia and early management may prevent serious complications.
